# Targeting and retention enhancement of quantum dots decorated with amino acids in an invertebrate model organism

**DOI:** 10.1038/srep19802

**Published:** 2016-01-25

**Authors:** Rui Xing, Xue-Dong Chen, Yan-Feng Zhou, Jue Zhang, Yuan-Yuan Su, Jian-Feng Qiu, Yang-Hu Sima, Ke-Qin Zhang, Yao He, Shi-Qing Xu

**Affiliations:** 1 School of Biology and Basic Medical Sciences, Medical College, Soochow University, Suzhou 215123, China; 2 National Engineering Laboratory for Modern Silk (NESER), Soochow University, Suzhou 215123, China; 3 Institute of Functional Nano & Soft Materials (FUNSOM), Soochow University, Suzhou 215123, China; 4 Research Center of Cooperative Innovation for Functional Organic/Polymer Material Micro/Nanofabrication, Soochow University, Suzhou 215123, China

## Abstract

The use of quantum dots (QDs) in biological imaging applications and targeted drug delivery is expected to increase. However, the efficiency of QDs in drug targeting needs to be improved. Here, we show that amino acids linked to CdTe QDs significantly increased the targeted transfer efficiency and biological safety in the invertebrate model *Bombyx mori*. Compared with bare QDs530, the transfer efficiency of Ala- and Gly-conjugated QDs (QDs530-Ala and QDs530-Gly) in circulatory system increased by 2.6 ± 0.3 and 1.5 ± 0.3 times, and increased by 7.8 ± 0.9 and 2.9 ± 0.2 times in target tissue silk glands, respectively, after 24 h of QDs exposure. Meanwhile, the amount of conjugated QDs decreased by (68.4 ± 4.4)% and (46.7 ± 9.1)% in the non-target tissue fat body, and the speed at which they entered non-target circulating blood cells significantly decreased. The resultant QDs530-Ala revealed a better structural integrity in tissues and a longer retention time in hemolymph than that of QDs530 after exposure *via* the dorsal vessel. On the other hand, QDs530-Ala significantly reduced the toxicity to hemocytes, silk gland, and fat body, and reduced the amount of reactive oxygen species (ROS) in tissues.

Due to their unique optical properties, aqueous synthesized quantum dots (aqQDs) are highly promising as fluorescent nano-probes for biological imaging applications, as well as a targeted drug delivery tool^1^,^2^,^3^,^4^,^5^,^6^. As well, quantum dots (QDs) are notable for their ability of potential targeted transfer in tissues, and even cells *in vivo*^7^,^8^. However, due to clearance of chemical groups on QDs’ surface by animal blood and nonspecific phagocytosis by the reticuloendothelial system (RES), the use of QDs is not yet a viable option for drug delivery^9^,^10^. One of the two significant challenges QDs-based drug delivery must overcome relates to the fact that targeted delivery of drugs/probes to tumors *in vivo via* the blood circulation system is often associated with nonspecific uptake by liver, spleen and other organs. It is difficult to effectively target and penetrate into the deep tissues of tumors^11^,^12^, and the amount of QDs entering tissues is low^13^. Additionally, researchers studying QDs-based drug delivery must consider that the effect of targeted drug delivery by QDs depends on *in vivo* QDs retention time. In addition to determining the efficiency of drug release, retention time is also the basis for intracellular imaging and therapeutic effects^14^,^15^,^16^. Therefore, effective targeting of tissues and extension of QDs retention time the circulatory system (hemolymph) are the two most urgent issues that must be resolved before QDs can be reliably used in targeted drug delivery.

A recent report showed that it is possible to reduce the clearance of QDs by phagocytes in mice by linking synthetic homologous polypeptides of human protein hCD47 to the QDs^17^. The *in vivo* targeted transport and biological characteristics of QDs modified by proteins, smaller polypeptides, or even amino acids is still an emerging field, and has far-reaching significance in biomedical and functional materials research^17^,^18^. This study focuses on linking single amino acid molecules, which are smaller than proteins or polypeptides, to the surface of QDs; the purpose of this study was to improve proper targeting by lowering the uptake of QDs in non-targeted tissues.

We chose to conduct our study using the silk gland tissues (SG) of silkworms *Bombyx mori*, due to their rapidly growing speed and powerful synthesis ability which similarities to some rapidly growing tumors. During the whole larval stage, the SG increases rapidly by 10 000 times and the cell metabolism extremely fast but no cell death, while nuclear DNA replication in SG cells is completed by several times and the DNA content increases 200000–400000 times within 3.5 weeks. The synthesized silk proteins in silk gland are more than 30% body weight of the organism. A silk gland synthesizes 6 × 10^9^ fibroin proteins per second, which is more than 60 times faster than the rate of a human liver cell synthesizing serum albumin^19^,^20^. In this study, in order to improve delivery to an assumed target organ- SG, single amino acids, which are needed in large amounts during protein synthesis in SG of the silkworm, were linked to the QDs under investigation. This study also investigated *in vivo* targeting efficiency in specific tissues and biological toxicity after oral exposure to QDs.

## The differences in tissue transfer ability of QDs in living organisms

**Figure 1:**
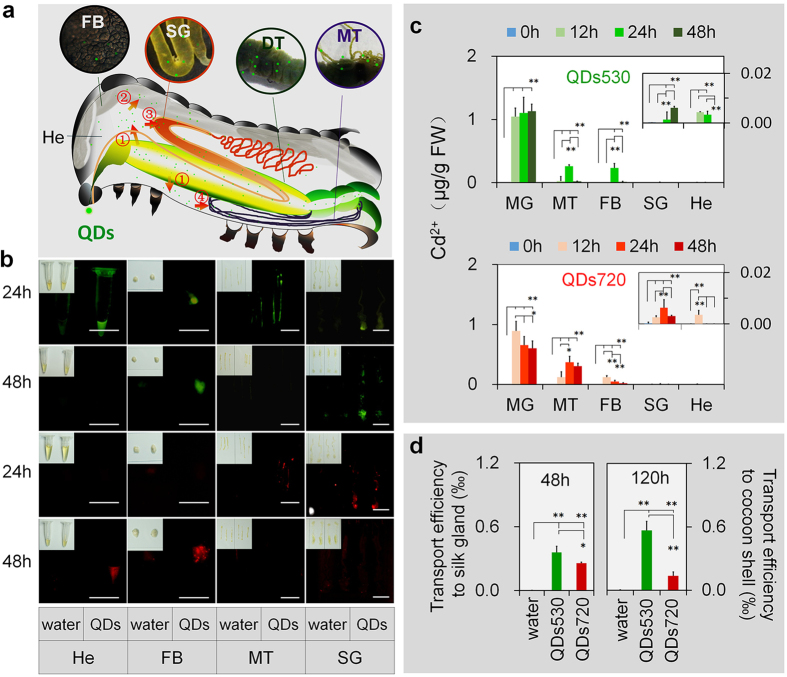
Test results of multiple tissues in silkworms. The 5th instar larvae 48 h after molting were injected with 0.56 nM (10 μl 56 μΜ) of CdTe QDs *via* the oral cavity. Exposure levels of QDs530 and QDs720, represented by Cd^2+^, were 60 μg and 65.3 μg per larva, respectively. The control organisms were injected with deionized water (water). (**a**) Basic transfer route of QDs among tissues *in vivo*. The QDs are injected orally and taken by the digestive track firstly. Subsequently, they pass through the digestive tract membrane barrier and arrive into the hemolymph (He)/blood cells. Finally, they are transported concomitantly to the floating tissues in hemolymph such as silk gland (SG), fat body (FB) and Malpighian tubule (MT). The arrow of number ① shows QDs from the digestive tract to hemolymph, the arrows of number ②,③ and ④ show QDs from hemolymph to the fat body, silk gland and Malpighian tubule concomitantly. (**b**) After 24 h or 48 h of exposure, the characteristic fluorescence of QDs was observed in multiple tissues at emission wavelengths of 530 nm and 720 nm. The green fluorescence indicated tissues with an accumulation of QDs530 and the red fluorescence indicated tissues with an accumulation of QDs720. The fluorescent images and the views of tissues were detected using CRI MaestroTM (Photometric, USA) and canon EOS70D camera (Cannon, Japan), respectively. The clean tissue removed peritrophic membrane from midgut (MG), which the longest part and main function part of the digestive tract (DT), was used to avoid the impact of food. (**c**) Atomic absorption spectrometry was used to determine the cadmium content in multiple tissues, (**d**) transport efficiency in silk glands 48 h after exposure, and in exocrine silk proteins 120 h after exposure. *P < 0.05 and **P < 0.01 indicate significant differences, every tissue sample was collected from 5 larvae and tested three times. Bars are 1 cm.

CdTe QDs passed through the digestive tract membrane barrier after 0.5 h of oral exposure. The tracing results on the outside of the larvae body showed that QDs720, whose particles are bigger in size, transferred faster from mouthparts to tail and had a better fluorescence effect (Fig. S1a). After 12 h of exposure, circulating blood cells (hemocytes) observed and the characteristic fluorescence of QDs was noted, confirming that QDs had entered the blood cells. Serious hemagglutination appeared in the QDs720 exposed group; this was not found in the QDs530-exposed group (Fig. S1b). These results indicated that the particle size of QDs directly affected their distribution of speeds and their status in the circulatory system (hemolymph); particle size may further affect QDs distribution in multiple tissues. The silkworm have an open circulatory system, all their organs just float in hemolymph which is a combination of lymph and blood cells (hemocytes). Figure 1a summarizes the QDs’ main probable transport route to the larval visceral tissues after oral exposure. The QDs are injected orally and taken by the digestive track firstly. Subsequently, they pass through the digestive tract membrane barrier and arrive into the hemolymph (He)/blood cells. Finally, they are transported concomitantly to the floating tissues in hemolymph such as SG and fat body (FB). Meanwhile, the QDs will be evacuated to Malpighian tubule (MT) in conjunction with water quickly. However, the most of water in MT will be reabsorbed by digestive track, while the QDs will be left in the urine and discharged into rectum from MT. The fat body (FB) are similar to human liver in the functions in biotransformation, although their organization forms are different.

Next, we focused on how the QDs were transported to visceral tissues. The characteristic fluorescence of QDs530 increased after 48 h of exposure to QDs530 in the fat body and silk glands. The increase of fluorescence indicated that QDs530 continuously accumulated in the tissues (Fig. 1b). Meanwhile, the characteristic fluorescence of QDs530 in excretory system Malpighian tubules and hemolymph almost disappeared after 48 h of exposure, indicating that either the amount of QDs530 was reduced, or QDs particles were destroyed (Fig. 1b). After 48 h of exposure to QDs720, changes in the accumulation and degradation of QDs720 in the fat body and Malpighian tubules were similar to those of QDs530. However, the changes to the QDs720 in hemolymph and silk glands were opposite of the changes experienced by QDs530, indicating that either the QDs530 entering the silk glands may remain there longer than QDs720, or that the QDs530 possesses a more stable particle structure (Fig. 1b).

The cadmium ion (Cd^2+^) content in tissues was determined, and the results are shown in Fig. 1c,d. The most notable results are as follows: 1) There were significant differences in the content of cadmium ions in different tissues. The highest Cd^2+^ content was in the midgut (MG), followed by the Malpighian tubules and fat body; blood and silk glands had very low Cd^2+^ content. MG is the best part and main function part of the digestive tract in silkworm larva. In this experiment, we used the cleaning tissue removed peritrophic membrane from midgut to avoid the impact of food. Moreover, the Cd^2+^ content in MT is no real content in the tissue cells but part is in the urine. Hence, we didn’t attention to midgut and Malpighian tubules. Instead, we focus on fat body; blood and silk glands. 2) The metabolic rates of QDs in tissues were related to particle size. QDs530 (smaller particle size) was metabolized slower and remained in the tissues longer. The content of QDs720 in the digestive tract began to decrease 24 h earlier than that of QDs530. The cadmium ion content of QDs530 in hemolymph, Malpighian tubules, and the fat body reached a peak 12 h later than QDs720 peaked. Furthermore, the presence of Cd^2+^ in excrement was determined 24 h after exposure. The content of Cd^2+^ in the QDs720 treatment group (1.82 ± 0.289 μg/g FW) was significantly higher than in the QDs530 treatment group (0.93 ± 0.166 μg/g FW). Cd^2+^ content was indicative of the amount of QDs present, and the silk gland was used as the target tissue to investigate the transfer efficiency of QDs. The results showed that the amount of QDs530 entering silk glands, and subsequently secreted with silk proteins to the outside of the silkworm, was significantly higher than QDs720. This indicated that QDs530, whose particles were smaller in size, were excreted slowly by the excretory system, thus remaining longer in the circulation system and target tissues.

In the body of silk worms, silk glands are specialized epidermal tissues. The toxicity of QDs entering the silk glands of 5th instar larvae mainly affected the growth of silk gland tissues and silk protein synthesis. On the contrast, it had very little direct impact on other vital visceral tissues and organs. This effect is QDs effectively impact on the target tissue silk gland, which similarities to the oncology drugs effect on the tumor tissues. Therefore, in follow-up experiments, we focused on enhancing the targeted transfer efficiency of the QDs to the silk gland tissues, as well as tissue toxicity analysis. These follow-up experiments were performed with the purpose of investigating the regulatory process of exogenous QDs transportation from the esophagus and hemolymph to gland cavities.

## Tissue-targeted transfer efficiency of QDs linked with amino acids

**Figure 2:**
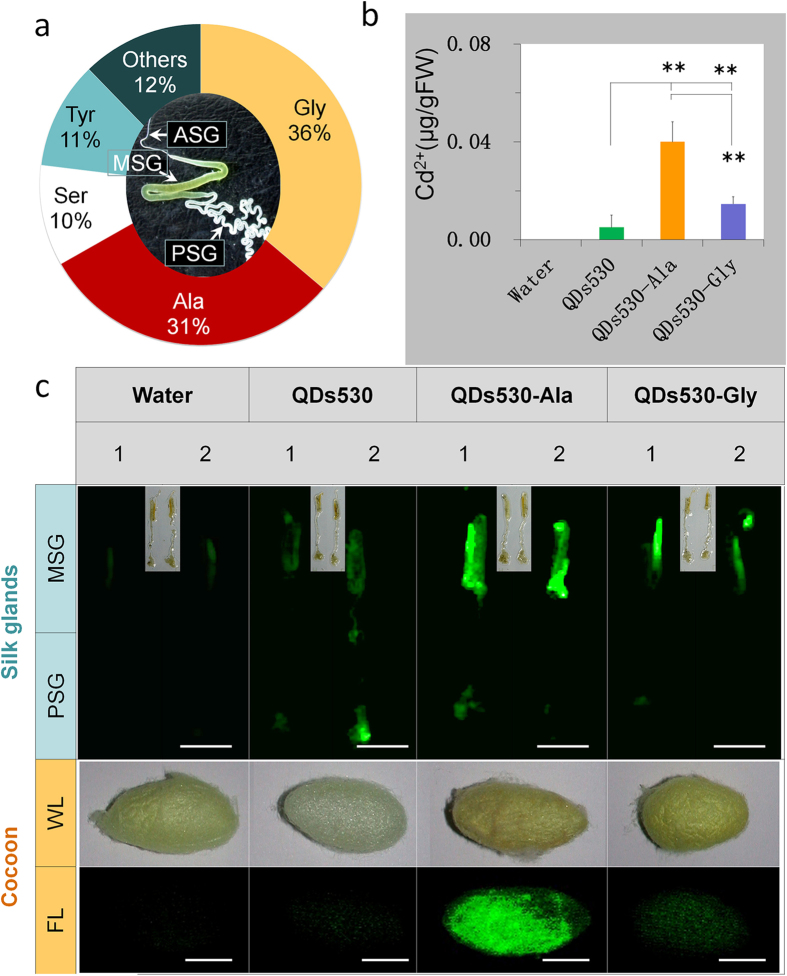
Transport efficiency of QDs linked with amino acids to silk glands. 0.56 nM of CdTe QDs was administered *via* oral injection; water was injected to control samples. (**a**) The external morphology of silk glands and the content of major amino acids in exocrine silk proteins synthesized by silk glands. (**b**) Cd ion content 24 h after exposure in multiple tissues, as measured by atomic absorption spectrometry. (**c**) Characteristic green fluorescence of QDs530 in silk glands 24 h after exposure, and in cocoons 168 h after exposure. *P < 0.05 and **P < 0.01 indicate significant differences, every tissue sample was collected from 5 larvae and tested three times. Bars are 1 cm.

Results show that the content of Ala and Gly accounted for up to 67% of the total amino acids in the exocrine silk proteins synthesized by silk glands, as seen in Fig. 2a. We use QDs coupled with alanine or glycine because these two amino acids are the most use for silk protein synthesis and possible to improve delivery to SG. Therefore, conjugated Ala and Gly to the surface of QDs530, forming QDs530-Ala and QDs530-Gly, respectively. QDs530 was chosen over QDs720 due its higher efficiency of entering silk glands, greater stability in tissues (Fig. 1), and lower toxicity to silk glands (Fig. S3). After oral exposure, results showed that the efficiency of entering silk glands was significantly enhanced for both amino acids-conjugated QDs. After 24 h of exposure, the contents of QDs530-Ala and QDs530-Gly in the silk glands, indicated by Cd^2+^, increased by 7.8 ± 0.9 fold and 2.9 ± 0.2 fold, respectively, compared with QDs530 content under similar conditions (Fig. 2b). Fluorescence observations of silk gland tissues and silk proteins (cocoons) secreted from exocrine silk glands proved that QDs530-Ala had very good structural stability (Fig. 2c). During silk protein synthesis in the silk glands, more Gly was needed than Ala, and the efficiency of QDs530-Ala entering silk glands was significantly higher than that of QDs530-Gly (Fig. 2). This indicated that in body tissues, there were significant differences in the absorption capacity of QDs linked with different amino acid molecules.

**Figure 3:**
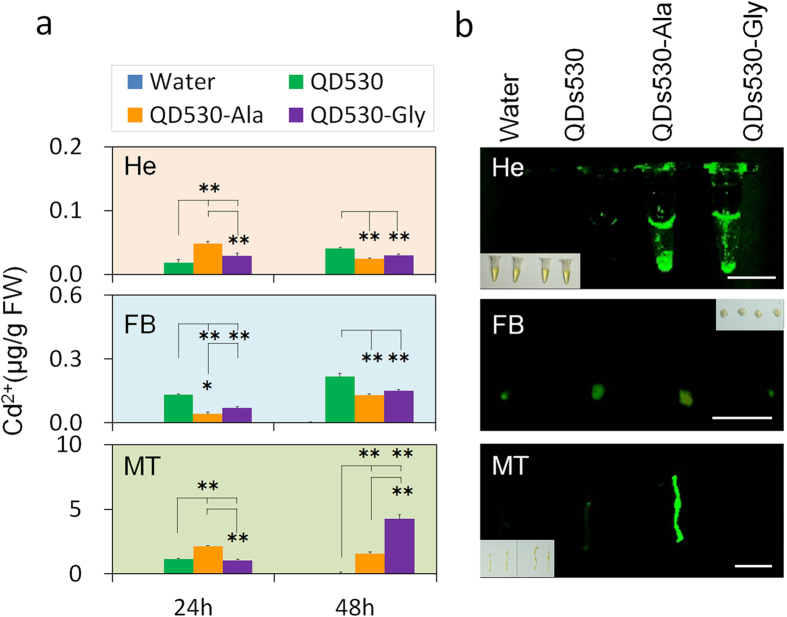
Changes in transfer efficiency of QDs530 linked with different amino acids between tissues *in vivo*. 0.56 nM of CdTe QDs was administered *via* oral injection; water was injected as a negative control. (**a**) Cd ion content in multiple tissues 24 h and 48 h after exposure, determined by the atomic absorption method. (**b**) Green fluorescence of QDs530 in multiple tissues was measured 24 h after exposure. He, hemolymph; FB, fat body; MT, Malpighian tubule; SG, silk gland; DT, digestive tract. *P < 0.05 and **P < 0.01 indicate significant differences, every tissue sample was collected from 5 larvae and tested three times. Bars are 1 cm.

Figure 3a summarizes the QDs’ main transport route to the silkworms’ visceral tissues after oral exposure. After entering the digestive tract *via* mouth, the QDs passed through the digestive tract inner membrane barrier and through the digestive tract tissues, and subsequently entered the hemolymph. The results shown in Fig. S1 prove that this process can be accomplished. Identifying them as foreign molecules, the Malpighian tubules in the excretory system excreted most of the QDs entering the hemolymph to the outside of the body (Fig. 1). Some QDs directly entered either the hemocytes (Fig. S1c), or silk glands, the fat body, and other tissues suspended in the hemolymph (Fig. 1).

It has been reported that uptake by non-specific tissues is one of the main reasons for reduced targeted transport of drugs/probes *in vivo* through the blood circulation system^11^,^21^. In order to ascertain the reasons for silk gland transport efficiency differences of the QDs modified with amino acids (shown in Fig. 2), we investigated the contents of QDs in hemolymph, Malpighian tubules of the excretory system and fat body tissues; fat body tissues have a similar storage function as silk glands. Furthermore, the accumulation differences of QDs530 with characteristic fluorescence were investigated in multiple tissues. The results in Fig. 3a,b show that 24 h after exposure, the amount of QDs530-Ala and QDs530-Gly in hemolymph increased 2.6 ± 0.3 fold and 1.5 ± 0.3 fold, respectively, compared with that of unlinked QDs530 (Fig. 3a). Characteristic fluorescence in hemolymph also showed significant improvements in the efficiency of Gly- and Ala-modified QDs530 at entering hemolymph after oral exposure (Fig. 3a). At 48 h after exposure, the contents of QDs530-Ala and QDs530-Gly in hemolymph were significantly lower than that of QDs530 (Fig. 3a). QDs content in Malpighian tubules showed that QDs linked with amino acids was excreted significantly slower through the excretory system (Fig. 3a). The Fig. 3b results showed that although the content of cadmium shown the QDs530 and QDs530-Gly existed in Malpighian tubules at 24 h after exposure to QDs, meanwhile the characteristic fluorescence were lost.

The results also showed that 24 h after exposure, the amount of QDs530-Ala and QDs530-Gly in the fat body was reduced to (68.4 ± 4.4)% and (46.7 ± 9.1)%, respectively, compared to QDs530 content. At 48 h after exposure, amino acid-modified QDs remaining in the fat body was significantly lower than the remaining QDs530 groups (Fig. 3a). The results presented in Fig. 3b also show that QDs530-Ala in the fat body had an intact particle structure, and its characteristic fluorescence intensity was higher than that of QDs530-Gly and QDs530.

## Biological toxicity of QDs linked with amino acids

Our previous studies have shown that vascular injection of sub-lethal doses of CdTe QDs (QDs530 or QDs720) in *B. mori* larvae caused time- and dose-dependent damage in the hematopoietic organ and hematocytes. QDs exposure promoted the mitotic nucleus in prohemocytes and hematocytes similar to peripheral blood stem cells in humans, but aggravated apoptosis^22^. In this experiment, in order to investigate the toxic variability of the QDs modifying with Ala or Gly, QDs530-Ala and QDs530-Gly were compared with the bare QDs530. These results indicated that modifying QDs with Ala and Gly can significantly reduce the QDs-induced blood cell apoptosis. The LD50 of QDs was obtained 72 h after oral exposure. The toxicity value was 3.96 nM (1.83–10.33 nM 95% confidence interval), which showed that the toxicity of QDs in the present study was lower than that in reference 22 *via* dorsal vein injection.

**Figure 4:**
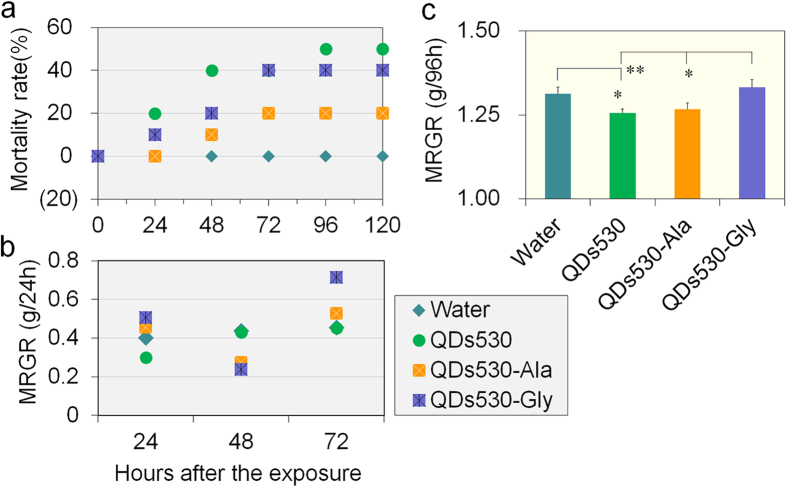
(**a**) Mortality (n = 10, treatment repeated 5 times). (**b**,**c**) Mean relative growth rate (MRGR) within 24 hours and 96 hours (n = 10, treatment repeated 3 times). 0.56 nM of CdTe QDs was administered to 5th instar larvae 48 h after molting *via* oral injection; water was injected as control. *P < 0.05 and **P < 0.01 indicate significant differences (n = 5 larvae and repeat test 3 times).

Death rates in the QDs530-Ala and QDs530-Gly group were significantly lower than that in the QDs530 group, and the time of death in the QDs530-Ala treated group was delayed (Fig. 4a). Toxicity of QDs530-Ala and QDs530-Gly on larval growth rates were significantly reduced in comparison with the toxicity of QDs530. The results of 24 h mean relative growth rate (MRGR) (Fig. 4b) showed that 72 h after exposure, the larval growth rates in the QDs530-Ala and QDs530-Gly group were restored. The results of 96 h MRGR (Fig. 4c) showed that the larval growth rate in the QDs530-Gly group was restored to the level of the negative control 96 h after exposure. The Fig. 4 results indicate a lower death rate for QDs530-Ala and little effect on the growth rate over long period of time.

**Figure 5:**
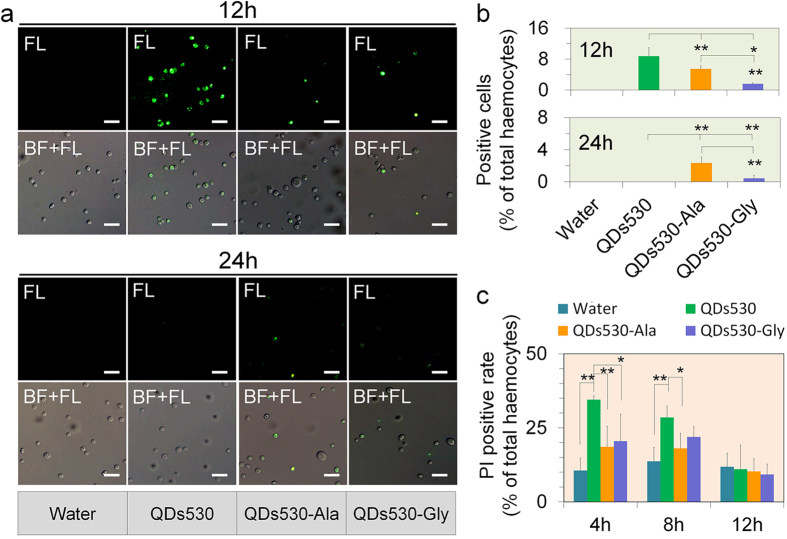
Amino acid modification changed the speed of QDs entering blood cells and their effect on apoptosis of blood cells. 0.56 nM of CdTe QDs was administered *via* oral injection; water was injected as a negative control. (**a**) Merged images for bright field and fluorescent light, revealing blood cells with accumulated QDs 12 h and 24 h after exposure. (**b**) The percentage of positive blood cells with QDs characteristic fluorescence in total blood cells at 12 h and 24 h after exposure. (**c**) Positive rate of PI staining of blood cells, indicating apoptosis of blood cells. The labels indicate: FL, fluorescent light; BF, bright field; BF + FL, merged images for bright field and fluorescent light, showing blood cells with accumulated QDs530. *P < 0.05 and **P < 0.01 indicate significant differences (n = 3). Bars are 10 μm.

The results shown in Fig. 4 indicate a lower death rate for QDs530-Ala and little effect on the growth rate over long period of time. Our previous studies have shown that vascular injection of sub-lethal doses of CdTe QDs (QDs530 OR QDs720) in *B. mori* larvae caused time- and dose-dependent damage in the hematopoietic organ and hematocytes. QDs exposure promoted the mitotic nucleus in prohemocytes similar to peripheral blood stem cells in humans, but aggravated apoptosis^22^. In this experiment, to further investigate the toxic variability of the QDs modifying with Ala or Gly, QDs530-Ala and QDs530-Gly were compared with QDs530. A body cavity injection of QDs was used to observe the speed at which QDs exposed directly to hemolymph entered blood cells, as well as the effects of QDs on apoptosis. This investigation was performed in order to assess the effects of amino acid modification on the toxicity of QDs. The results presented in Fig. 5a,b show that, compared to treatment with QDs530, the proportion of hemocytes displaying the QDs’ characteristic green fluorescence 12 h after exposure decreased significantly for larvae treated with QDs530-Ala or QDs530-Gly, with the QDs530-Gly treated group showed a larger decrease. The proportion of positive blood cells with the characteristic green fluorescence of QDs530 in the hemocytes of all QDs-treated larvae dropped significantly 24 h after exposure, compared with that of larvae 12 h after exposure (Fig. 5b). Almost no positive blood cells were found in the QDs530-treated group, while positive blood cells were still observed in the QDs530-Ala and QDs530-Gly treated groups. However, the fluorescence intensity of positive blood cells in the QDs530-Gly treated group was weaker (Fig. 5a). These results further demonstrated that the retention time of Ala- and Gly-modified QDs530 was extended in circulating hemolymph, but the speed at which they entered hemocytes was slow. QDs530-Ala maintained its characteristic fluorescence and an intact particle structure better than QDs530-Gly and QDs530.

Apoptosis of blood cells in circulating blood was further determined. The results presented in Fig. 5c show that, compared with the control, the positive rate of propidium iodide staining (PI) of blood cells increased for the QDs-injected samples 4 h and 8 h after the body cavity injection. Meanwhile, the positive rate of PI staining in the QDs530-Ala treated group decreased significantly compared with the QDs530 treated group; the positive rate of PI staining in the QDs530-Gly treated group was also significantly lower than that of QDs530 4 h after exposure. These results indicated that modifying QDs with Ala and Gly can significantly reduce QDs-induced blood cell apoptosis. However, all samples same apoptosis after 12 h, it is possible that hematopoietic recovery of hematopoietic organs (Hos) after QDs exposure took place within a certain concentration range^22^.

**Figure 6:**
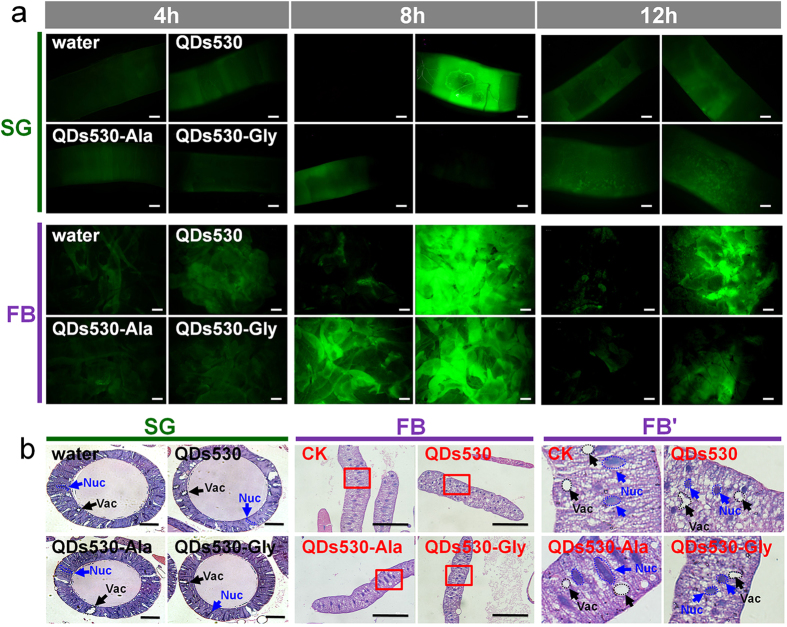
(**a**) Reactive oxygen species (ROS) levels in target tissue silk glands (SG) and metabolic tissue of the fat body (FB) at 4 h, 8 h and 12 h after exposure to QDs and (**b**) Hematoxylin-eosin (HE) staining of tissue morphology at 24 h after exposure. 0.56 nM of CdTe QDs was administered *via* oral injection. Water was injected as a negative control. FB’ is a partial enlarged view of FB. Nuc, nucleus. Vac, vacuolation. Bars are 100 μm.

ROS induced by QDs is an important mechanism in their cellular and biological toxicity^23^. The results in Fig. 6a show that in the silkworm larvae, a large number of ROS were generated by QDs530 in the metabolic tissue of the FB and the exocrine tissue of the SG within 8 h after oral exposure. ROS in these two tissues began to decrease 12 h after exposure, after which the ROS in the SG almost not shown and recovered to the level of the negative control. ROS production in the FB and SG exposed to amino acid-modified QDs530 was significantly lower than in larvae exposed to QDs530 during the corresponding period. ROS levels in the SG and FB at 12 h after exposure to QDs530-Ala were restored to the level of the control group. Changes in tissue morphology in the SG and FB were observed 72 h after exposure. QDs did not cause obvious tissue damage and morphological variation in the SG, but QDs-induced cell damage occurred in the FB (Fig. 6b). In the FB tissue cell nuclei, apoptotic changes appeared 48 h after exposure to QDs, but the degree of apoptotic changes in these cell nuclei decreased when Ala or Gly was linked to the QDs; almost no difference was observed between the QDs530-Ala group and the control group (Fig. 6b). Observational results of intracellular lysosomal autophagy repair for damages caused by QDs-induced ROS^24^ also proved that a large number of lysosomes appeared in FB cells as a result of QDs exposure. Meanwhile the number of lysosomes in FB cells decreased when silkworms were exposed to Ala- or Gly-linked QDs; the number of lysosomes in the QDs530-Ala exposure group was close to the control (Fig. S4a). It is significant to note that there were almost no lysosomes in SG cells, showing that this tissue carries out autophagy repair almost completely independent of lysosomes (Fig. S4b). This also supports the observed results of undamaged SG tissues in Fig. 6b.

The above results showed that the toxicity of QDs to the silkworms’ exocrine tissue in the SG was significantly lower than that in the metabolic tissue of the FB. Amino acids-modified QDs-induced ROS production in visceral tissues decreased, thus reducing the toxicity of QDs, especially QDs530-Ala.

## Conclusion

Ala, an amino acid needed in a large number in target tissue silk glands, was linked to QDs530. This modification increased the efficiency of QDs entering the hemolymph *via* oral exposure, extended the retention time in circulation system, and reduced the efficiency of entering non-target metabolic tissue in the fat body. This significantly increased the transfer efficiency to silk glands, maintained the nanoparticle structure of QDs, and significantly reduced the toxicity of CdTe QDs to hemocytes, metabolic organization fat body, and even organism. In QDs530 linked with Gly, another amino acid required in a large number of target tissue silk glands, the transfer efficiency to target tissue silk glands decreased, and toxicity to tissues and organs was greater than that of QDs530-Ala. This might be related to the clearance of QDs530–Gly nanoparticles faster than that of QDs530–Ala. The structural stability of QDs is related to the shells^25^,^26^. Our results showed that although the kinetics of transport based on content of cadmium shown the QDs existed in some tissues, meanwhile their characteristic fluorescence were lost, such as in the hemolymph at 24 h after exposure to QDs720 or in Malpighian tubules after exposure QDs530-Gly. The absence of fluorescence in can be either due to destruction of the QDs fluorescent properties or to the QDs having left the tissues but the former remains. Since the existence of cadmium was virtually undetectable in control silkworm tissues. The biggest cause of inconsistency was that the destruction of QDs530-Gly fluorescent properties ahead of QDs530-Ala in the excretory systems, and same as the QDs720 ahead of QDs530 in the circulation systems.

The results of the present study suggest that a more efficient method of QDs transfer in tissues may be established. Furthermore, the use of nanoparticles in drug delivery and imaging may be improved by identifying the responses of different animal (and human) tissues to various amino acids. Thus, the effectiveness of employing nanoparticles in drug delivery can be increased by advantageously applying different rates of amino acid absorption to varying tissues, or using single organic molecules distinctive from normal tissue cells, which are needed in fast-growing tumor cells. When performing these procedures, one should carefully regard *in vivo* QDs stability of different animals, as well as assess the biological safety of studies linking other types of amino acids or other single molecules to QDs for improvement of targeted transfer efficiency.

## Methods

### CdTe quantum dots

Two sizes of water-soluble CdTe quantum dots with maximum luminescent wavelengths of 530 nm (QDs530) and 720 nm (QDs720) were used in these experiments. Moreover, two types of QDs530 with negative coatings coated with amino acids Ala (QDs530-Ala) or Gly (QDs530-Gly) were used, too. These QDs were synthesized based on previously reported methods^18^,^27^. To summarize the process, QDs precursor solution was prepared by adding freshly prepared NaHTe solution to N_2_- saturated CdCl_2_ solution at pH = 8.4, in the presence of a stabilizer, 3-mercaptopropionic acid (MPA). Controlling the reaction time and microwave irradiation (MWI) temperature allowed for variation in the maximum luminescent wavelengths of different QDs. Here, QDs530, and QDs720 were synthesized at 100 °C/5 min and 160 °C/10 min, respectively. In order to exclude residual reagents such as MPA, Cd^2+^, and Te^2+^, the as-prepared CdTe solution was concentrated to a quarter of the original volume. Then, the aqQDs was precipitated with 2-propanol and collected *via* centrifugation. The carboxylic acid groups of QDs was made to readily react with the amino groups of the amino acid by using N-(3-dimethylaminopropyl)-N-ethylcarbodiimde hydrochloride (EDC) as zero-length cross linkers^28^,^29^. In brief, 25 μl EDC (10 mg/ml) was first added to 300 μl QDs, with the mixed solution incubated at 25 °C for 20 min to fully activate the QDs. Following this, 5 μl Ala or Gly (5 mg/ml) solution was added to the activated QDs, and the resultant solution was incubated for 2 h at 25 °C under shaking in dark. To exclude the unreacted amino acid, the resultant solution was filtrated using 10 KDa Nanosep centrifugal devices three times, at 6000 rpm for 15 min, and then stored at 4 °C in dark for the following experiments.

### Preparation of the test organism

Larvae of *B. mori Dazao* strain were reared on fresh mulberry leaves at 25 °C with a photoperiod of 12 h light and 12 h dark, then preprocessed following the method of Liu *et al.* (2014)^20^. Similar-sized larvae (1.403 ± 0.098) g in the 48 h of the 5^th^ instar were exposed to QDs. In the early stage, lethal toxicity studies were performed in order to assess the animal’s tolerance and to accurately set the sub-lethal doses of QDs. 0.56 nΜ (56 μM × 10 μl) QDs per individual were selected to assess the effect on the targeting drug system (TDS), and control organisms were injected with DI water. The route of administration was oral and vascular injection. After exposure, body weight and survival of the larvae were recorded every 12 h. Mortality and mean relative growth rate (MRGR) were also determined. For larvae that developed to the spinning stage, cocoon shells and silkworm bodies were weighed 72 h after spinning, and silk protein secretion efficiency was calculated.

### Investigation of tissue distribution speed and transport efficiency of QDs

Hemolymph was collected after the anal horns of silkworms were cut following 4–72 h of QDs exposure. The silkworm body was quickly damaged, and the alimentary canal, Malpighian tubules, fat body and silk glands were separated and washed with pre-cooled NS; fluorescence was detected immediately following this procedure. The characteristic fluorescence of QDs was detected at the emission wavelengths of 530 nm (QDs530, green fluorescence) and 720 nm (QDs720, red fluorescence) using CRI MaestroTM (Photometric, USA). Resampling was performed to determine cadmium ion content in the tissues. Tissue samples were repeatedly weighed and digested, and cadmium ion contents were determined by atomic absorption spectrometry (AA240FS-GTA120, Perkin Elmer, USA). Tissue transfer efficiency of QDs was represented by the ratio of cadmium ion content in tissues to the total amount of exposed cadmium ion.

### Tissue staining

Following the method of Liu *et al.* (2014)^22^, the hemocytes were stained with Propidium Iodide (PI) (Invitrogen, USA) in order to assess cell apoptosis, and the levels of reactive oxygen species (ROS) were measured using a ROS kit (Beyotime, Jiangsu, China) in order to assess degree of oxidative stress. For the sake of contrast tissue morphology, the target tissue silk glands and the non-targeted tissue of fat body were isolated on ice and fixed in 4% paraformaldehyde. Then the tissues were made of paraffin sections and were further stained by Hematoxylin and Eosin (HE) (Beyotime, Jiangsu, China) performed as described by Ji *et al.* (2013)^19^.

### Determination of amino acid levels

The level of amino acids in silk glands were measured by pre-column derivatization of HPLC (Ag1100, Agilent, Palo Alto, USA) using a ODS HYPERSIL column (250 × 4.6 mm, 5 μm). Silk glands were quickly dissected from larvae. Then 0.5 g of silk glands were weighed and ground with liquid nitrogen. 1 ml of pure water was added to the mixtures which were then transferred into EP tubes. The mixtures were homogenized with a cell disrupter for 1 min. Finally, the mixtures were centrifuged at 4 °C and 12000 rpm for 10 min and the supernatant was stored at −80 °C until use.

## Additional Information

**How to cite this article**: Xing, R. *et al.* Targeting and retention enhancement of quantum dots decorated with amino acids in an invertebrate model organism. *Sci. Rep.* **6**, 19802; doi: 10.1038/srep19802 (2016).

## Supplementary Material

Supplementary Information
