# Supplementary material for: Targeting and retention enhancement of quantum dots decorated with amino acids in an invertebrate model organism
Source: Sci Rep. 2016 Jan 25;6:19802. doi: 10.1038/srep19802 (PMC4726310; doi:10.1038/srep19802)
Supplement: Supplementary Information [file srep19802-s1.doc]

Targeting and retention enhancement of quantum dots decorated with amino acids in an invertebrate model organism

Rui Xing^1,2,🞤^, Xue-Dong Chen^1,2,🞤^, Yan-Feng Zhou^3,🞤^, Jue Zhang^2^, Yuan-Yuan Su^3^, Jian-Feng Qiu^1,2^, Yang-Hu SiMa^1,2^, Ke-Qin Zhang^2,4,🞸^, Yao He^3, 🞸^, Shi-Qing Xu^1,2,🞸^

1 School of Biology and Basic Medical Sciences, Medical College, Soochow University, Suzhou 215123, China.

2 National Engineering Laboratory for Modern Silk (NESER), Soochow University, Suzhou 215123, China.

3 Institute of Functional Nano & Soft Materials (FUNSOM), Soochow University, Suzhou 215123, China.

4 Research Center of Cooperative Innovation for Functional Organic/Polymer Material Micro/Nanofabrication, Soochow University, Suzhou 215123, China

^🞸^ Corresponding author: kqzhang@suda.edu.cn, yaohe@suda.edu.cn, szsqxu@suda.edu.cn

^🞤^ These authors contributed equally to this work.

Supplementary Information

**Particle size of CdTe QDs *via* oral exposure affected the speed of distribution in *B. mori***

Silkworm larvae were exposed to 0.56 nM (10 μl 56 μM) QDs530 *via* oral administration. The characteristic green fluorescence of QDs530 was observed on the outside of the body after 4 h of exposure, and fluorescence intensity increased up to 24 h of exposure. Fluorescence intensity at 48 h was weaker than that at 24 h (Fig. S1a). The characteristic red fluorescence was clearly observed on the outside of the body after exposure to QDs720 for 0.5 h. Fluorescence mainly appeared in the front and central part of the body within 4 h of exposure; fluorescence occurred in the central and rear part of the worm at 12 h. Fluorescence was distributed throughout the body at 24 h; red fluorescence almost disappeared at 48 h (Fig. S1a). The characteristic fluorescence of QDs observed on the outside of the body indicated that QDs *via* oral administration passed through the peritrophic membrane barrier of the digestive tract into the hemolymph. Disappearance of the characteristic fluorescence of QDs observed on the outside of the body suggested that QDs in hemolymph were transported to the visceral tissues suspended in hemolymph or excreted by the excretory system. It is also possible that the nanoparticles were directly destroyed in the hemolymph.

Further observation of hemocytes was performed. The characteristic green fluorescence of QDs530 and the characteristic red fluorescence of QDs720 appeared in a certain proportion of hemocytes 12 h after exposure to QDs (Fig. S1b), demonstrating that water-soluble CdTe QDs enter blood hemolymph *via* oral exposure, through the digestive tract barrier. Similar results were also confirmed in *Drosophila* and other organisms treated with CdSe-ZnS QDs^S1^. Serious blood cell hemagglutination was found after oral exposure to QDs720, which was not found after exposure to QDs530 (Fig. S1b). The results in Fig. S1 show that the particle size of QDs directly affected QDs transport speed in the hemolymph, and may have affected their further distribution in multiple tissues.


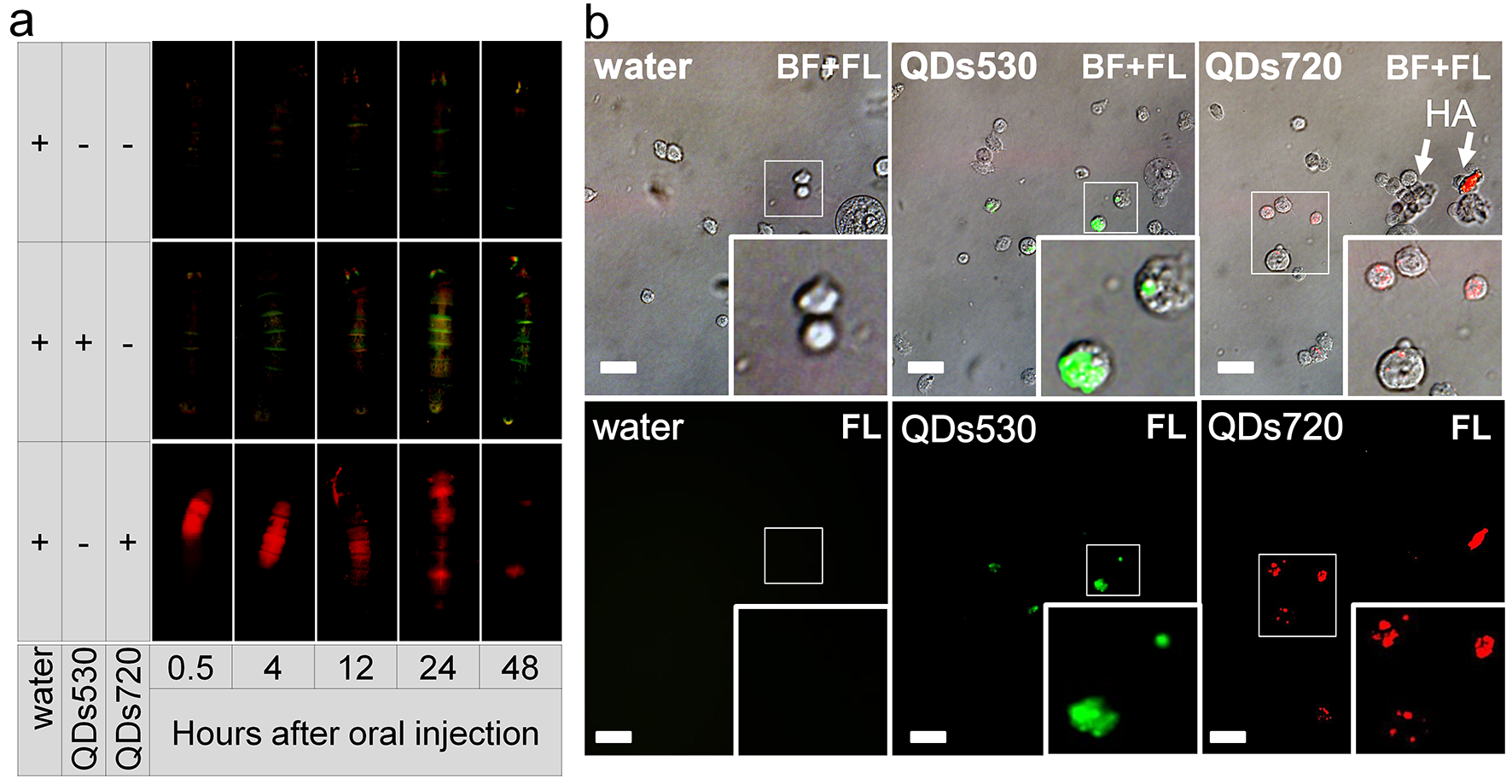


**Figure S1. (S1a) Changes in the distribution of the characteristic fluorescence of CdTe QDs530 and QDs720 in organisms after oral exposure and (S1b) the results in hemocytes 12 h after exposure.** 0.56 nM of CdTe QDs was administered *via* oral injection to 5th instar larvae 48 h after molting; the same volume of water was injected as a negative control. The labels indicate: FL, fluorescent light; BF, bright field; BF + FL, merged images for bright field and fluorescent light, showing QDs-enriched blood cells. HA, Hemagglutination. QDs530 displayed green fluorescence at an excitation wavelength of 490 nm and emission wavelength of 530 nm, while QDs720 showed red fluorescence at an excitation wavelength of 670 nm and emission wavelength of 720 nm. Bars are 10 μm.

**Preparation of CdTe quantum dots**

The as-prepared QDs were first confirmed by UV-vis absorption spectra, as displayed in Fig. S2a. The normalized photoluminescence (PL) spectra of the QDs are shown in Fig. S2b. As shown in Fig. S2c, the corresponding hydrodynamic diameters were 3.6 and 8.7 nm for QDs530 and QDs720respectively. Fig. S2d and S2e show high-resolution transmission electron microscope (HRTEM) images of QDs530 and QDs720, which were 2.8 and 3.6 nm, respectively. The slight deviation in diameter measured by dynamic-light-scattering (DLS) and transmission electron microscope (TEM) is attributed to different surface states of the samples under the tested conditions, which was discussed in our previous work^S2, S3^. The corresponding Fourier transform infrared spectroscopy (FTIR) absorption spectra of the QDs530 and QDs530-AA (Ala or Gly) are shown in Fig. S2f.


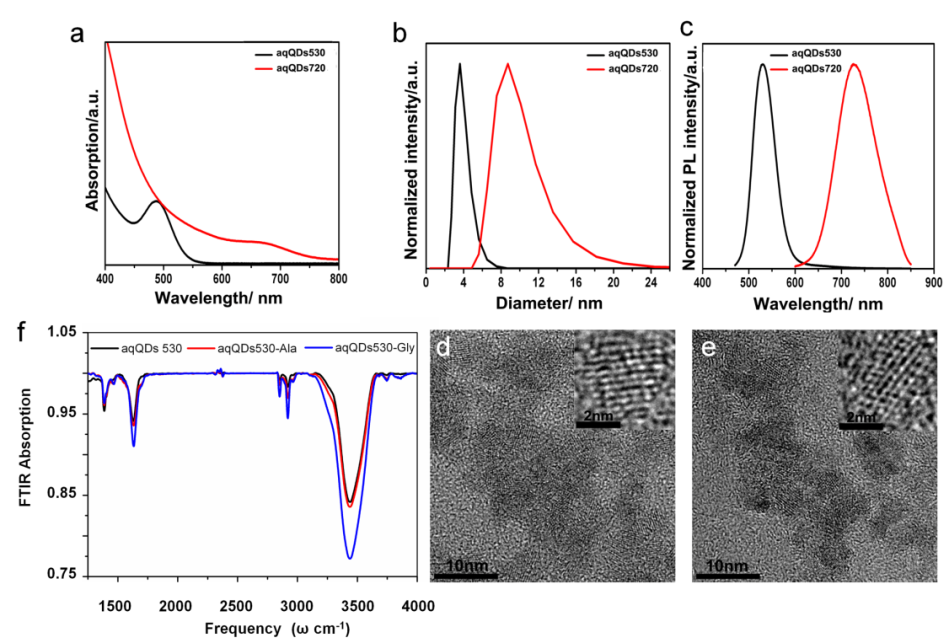


**Figure S2.** (S2a)Absorption and (S2b) photoluminescence spectra of the different-sized QDs used in our study, whose maximum luminescent wavelength was 530 nm (QDs530), and 720 nm (QDs720), respectively. (S2c) A representative dynamic-light-scattering histogram of the QDs. Their corresponding transmission electron microscope (TEM) images are shown in figure S2d and S2e. Figure S2f shows the corresponding Fourier transform infrared spectroscopy (FTIR) absorption spectra of QDs530 and QDs530-AA (Ala or Gly). Notably, we found that the absorbance at 1600–1680, 2800–3000 and 3400 cm^-1^ were increased after QDs530 were decorated with Ala or Gly. Absorbances at 1600-1680, 2800-3000 and 3400 cm^-1^, which were assigned to the C=O bonds (stretching vibration), O-H bond (stretching vibration) and N-H bonds (stretching vibration), respectively, indicated abundant carboxylic acid groups and amino bonds in the prepared QDs530-AA (Ala or Gly).

**Particle size of QDs was related to toxicity in target tissues**

Following oral administration of the QDs, QDs530, which entered the silk glands had no adverse effects on silk protein production, while QDs720 reduced silk protein synthesis and exocrine capacity in silk glands (Fig. S3a). The mechanical properties of silk fibers were measured using Instron5967 (Instron, USA). The results showed that after exposure to QDs530 and QDs720, although elongation (Fig. S3b) and the breaking strength (Fig. S3c) of the first 100 m of silk fibers secreted did not differ from those of the control, elongation of the subsequent 200-300 m of silk fibers secreted (Fig. S3b) was greater than that of the control. The breaking strength of the 200-300 m of silk fibers in the QDs530 treated group was also higher than that of the control (Fig. S3c).


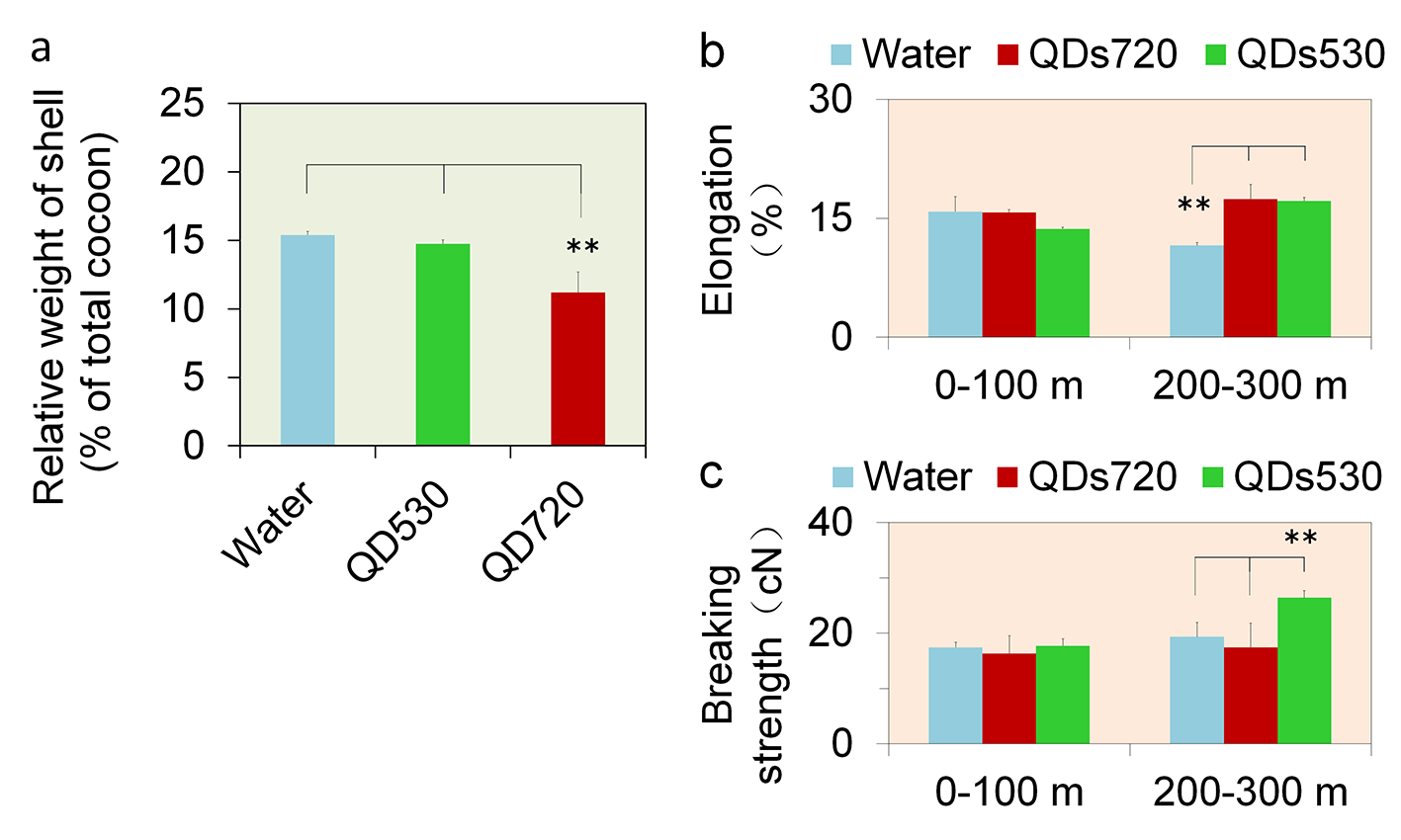


**Figure S3. Effect of QDs *via* oral administration on secretion efficiency of (S3a) silk proteins in silk glands 120 h later, (S3b) elongation and (S3c) breaking strength of silk fibers.** 0.56 nM of CdTe QDs was administered to 5th instar larvae 48 h after molting *via* oral injection, and water was injected as a negative control. ** *P* <0.01 indicate significant differences (n=3).

**Staining of lysosomes in tissue cells**

The toxicity of QDs-induced ROS, heavy ions and other direct QDs impact on nuclei result in cytotoxicity, inducing autophagy and death^S4^. Lysosomes play an important role when threats to the nuclei appear^S5^. The results of lysosomal staining in silkworms, following the methods developed Tian et al. (2013)^S6^, showed that a large number of lysosomes appeared in FB cells after exposure to QDs, while the number of lysosomes in FB cells significantly reduced after exposure to QDs linked with Ala or Gly. The QDs530-Ala exposure group showed a more obvious effect, which was close to that of the control group (Fig. S4a). Almost no lysosomes appeared in the SG cells (Fig. S4b).


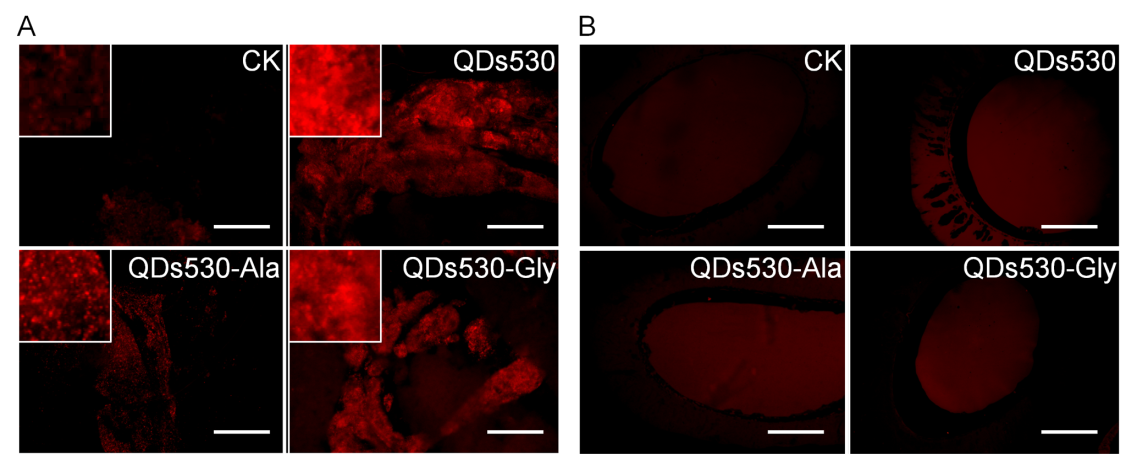


**Figure S4. (S4a) Staining results of lysosomes in fat body and (S4b) silk gland tissues after 24 h of exposure** (Lyso-Tracker Red, Beyotime C1046, China). 0.56 nM of CdTe QDs was administered to 5th instar larvae 48 h after molting *via* oral injection, and water was injected as a negative control. Bars are 50 μm.

**References**

S1. Galeone, A., et al. *In vivo* assessment of CdSe-ZnS quantum dots: coating dependent bioaccumulation and genotoxicity. *Nanoscale*. **4**, 6401-6407 (2012).

S2. He, Y. et al. Microwave-assisted growth and characterization of water-dispersed CdTe/CdS core-shell nanocrystals with high photoluminescence. *J Phys Chem B*. **110**, 13370-13174 (2006).

S3. He, Y., et al. Microwave synthesis of water-dispersed CdTe/CdS/ZnS core-shell-shell quantum dots with excellent photostability and biocompatibility. *Adv Mater*. **20**, 3416-3421 (2008).

S4 Winnik, F. M., Maysinger D. Quantum dot cytotoxicity and ways to reduce it. *Acc Chem Res*. **46**，672-680 (2013).

S5 Moreau, K., Luo, S. Q. & Rubinsztein, D. C. Cytoprotective roles for autophagy. *Curr Opin Cell Biol*. **22**, 206-211 (2010).

S6 Tian, L., et al. 20-hydroxyecdysone upregulates Atg genes to induce autophagy in the *Bombyx* fat body. *Autophagy*. **9**, 1172-1187 (2013).
